# Medidas Hipertensivas em Escolares: Risco da Obesidade Central e Efeito Protetor da Atividade Física Moderada-Vigorosa

**DOI:** 10.36660/abc.20180391

**Published:** 2020-07-28

**Authors:** Tatiana Affornali Tozo, Beatriz Oliveira Pereira, Francisco José de Menezes, Cristianne Morgado Montenegro, Carla Marisa Maia Moreira, Neiva Leite

**Affiliations:** 1 Universidade do Minho Braga Portugal Universidade do Minho, Braga - Portugal; 2 Universidade Federal do Paraná CuritibaPR Brasil Universidade Federal do Paraná, Curitiba, PR - Brasil; 3 Universidade do Porto Porto Portugal Universidade do Porto, Porto - Portugal

**Keywords:** Criança, Adolescente, Escolares, Atividade física, Circunferência da Cintura, Índice de Massa Corporal, Hipertensão, Pressão Arterial

## Abstract

**Fundamento:**

Aumento da prevalência de hipertensão arterial (HA) em crianças e adolescentes e sua associação com diversas comorbidades.

**Objetivo:**

Verificar a associação de HA, obesidade central e obesidade geral, e nível de atividade física em escolares.

**Métodos:**

Participaram do estudo 336 crianças e adolescentes, de 11 a 17 anos de idade. Aferiram-se estatura, peso corporal, circunferência da cintura (CC) e pressão arterial (PA). Foi calculado o índice de massa corporal escore z (IMC-z). O nível de atividade física foi avaliado pela versão curta do *International Physical Activity Questionnaire* (IPAQ), conforme a prática em atividades físicas moderadas-vigorosas (AF-mv). Consideraram-se hipertensos os escolares que apresentaram pressão arterial sistólica (PAS) e/ou diastólica (PAD) superiores ao percentil 95, de acordo com sexo, faixa etária e estatura, ou ≥120×80mmHg. Utilizaram-se os testes estatísticos de *t-Student* , Qui-quadrado, Mann-Whitney e modelo de regressão logistica binária, considerando-se o nível de significância de p<0,05.

**Resultados:**

Foram observados que 40,5% dos escolares apresentaram HA, 35,11% excesso de peso (12,5% obesos), 13,39% CC elevada e 40,2% foram considerados insuficientemente ativos em AF-mv. As chances de HA foram relacionadas à CC elevada (OR=6,11; IC95%:2,59 a 14,42) e ao excesso de peso (OR=2,91; IC95%:1,76 a 4,79). Além disso, os adolescentes que praticavam AF-mv apresentaram menor risco de PAD elevada (OR=0,33; IC95%:0,15 a 0,72).

**Conclusão:**

Concluiu-se que a obesidade central, a obesidade geral e o sexo masculino foram os melhores preditores de HA em crianças e adolescentes. A prática de AF-mv demonstrou efeito protetor na PAD elevada em escolares. (Arq Bras Cardiol. 2020; 115(1):42-49)

## Introdução

A frequência de HA aumentou em todas as faixas etárias e em diversos países,^1^ atingindo crianças e adolescentes e tende a persistir ao longo do tempo, com a probabilidade elevada de progredir na vida adulta,^2^ principalmente pela crescente prevalência da obesidade,^3^ que está associada ao aparecimento de diversas comorbidades.^4^

A análise conjunta dos hábitos de vida que podem predispor ao aparecimento de doenças cardiovasculares na idade adulta tem papel importante para prevenção da hipertensão em crianças e adolescentes.^5^ A obesidade apresenta origem multifatorial, envolvendo aspectos do comportamento relacionados a alimentação, atividade física e fatores psicológicos.^6^

Portanto, o diagnóstico precoce da HA em crianças e adolescentes é relevante para se evitar o avanço da doença para a fase adulta, reduzir o risco de problemas cardiovasculares^7^ e recomendar programas terapêuticos para reverter esse processo.^8^ Dessa forma, medidas antropométricas como o índice de massa corporal (IMC) e a CC são indicadores de baixo custo e eficientes para identificar riscos cardiovasculares.^9^ O IMC classificado como excesso de peso demonstra obesidade geral,^10^ e a CC aumentada relaciona-se à obesidade central;^11^ esta última apresenta maior associação ao processo inflamatório em adultos e aparecimento das comorbidades cardiometabólicas.^12^

A mensuração da PA deve ser realizada em três ocasiões diferentes^13^ para confirmar o diagnóstico de HA, mas em estudos epidemiológicos geralmente é realizada em um dia e tem sido utilizado o termo “medidas hipertensivas”.^14^ Portanto, em crianças e adolescentes, alguns estudos demonstram maiores associações entre HA, obesidade central^15^ e obesidade geral,^16^ o que gera controvérsias entre a localização da adiposidade e a PA nesta população.

Além disso, as evidências sobre a prática AF-mv e PA ainda são limitadas, bem como a relação entre medidas antropométricas e AF-mv como protetores de HA em crianças e adolescentes. Dessa forma, é importante identificar o risco de HA na adolescência para a prevenção do avanço dessa condição na vida adulta, o que pode aumentar a eficiência do tratamento. Portanto, este estudo tem como objetivo verificar a associação entre AF-mv e indicadores antropométricos de obesidade com o diagnóstico de HA em crianças e adolescentes.

## Métodos

Realizou-se um estudo descritivo transversal quantitativo na cidade de São José dos Pinhais, Paraná (região Sul do Brasil). A amostragem foi constituída por conglomerados, escolhidos por conveniência (cada escola particular dos ensinos fundamental e médio da cidade foi considerada um conglomerado). Das seis instituições convidadas, localizadas na região central, somente duas escolas particulares aceitaram participar do estudo, em que foram convidados todos os escolares dos ensinos fundamental e médio.

No município, foram matriculados aproximadamente 55.289 estudantes nos níveis de ensino fundamental, nas séries finais, e ensino médio no ano de 2018.^17^ Estipulou-se a prevalência de 12,5% de crianças e adolescentes hipertensos na região sul do Brasil.^18^ Com base na seleção amostral probabilística, obteve o número total de 111 adolescentes para inferência da população estudantil na faixa etária estipulada. Incluiu-se 1,5x de indivíduos referente ao efeito de delineamento, levando-se em consideração o erro amostral de 5%, sendo incluídos mais 30% em virtude de possíveis desistências, resultando em um total de 217 indivíduos entre 11 e 17 anos de idade.

Participaram do estudo 336 crianças e adolescentes voluntários, com idade de 11 a 17 anos, de ambos os sexos (173 meninas e 163 meninos). Foram excluídos do estudo gestantes, indivíduos com limitações que os impedissem de participar de algum procedimento do estudo e aqueles que não tiveram o Termo de Consentimento Livre e Esclarecido (TCLE) e o Termo de Assentimento Livre e Esclarecido (TALE) assinados. Todos os procedimentos foram aprovados pelo Comitê de Ética em Pesquisa da Pontifícia Universidade Católica do Paraná, PUC – PR, CAAE (71324017.1.0000.0020/2017.)

As medidas antropométricas foram coletadas na escola, de maneira padronizada, seguindo os procedimentos preconizados pelo *Anthropometric Standartization Reference Manual* .^19^ A estatura foi aferida por meio de um estadiômetro portátil, com resolução de 0,1 centímetro (cm), e expressa em cm. O peso corporal foi avaliado com uma balança portátil modelo PLENA, com resolução de até 100 gramas e capacidade de 150kg.

Calculou-se o IMC-z,^20^ por meio do *software* WHO Anthro Plus®, versão 1.0.4. Classificaram-se como eutróficos os participantes com IMC-z entre ≥−2 e <+1, com sobrepeso entre ≥1 e <2, e obesos aqueles com ≥2; de acordo com a faixa etária e o sexo, foram considerados com obesidade geral os adolescentes classificados como sobrepeso e obesidade (IMC-z ≥1). Para a mensuração da CC, foi utilizada uma fita métrica inelástica, no ponto médio entre o último arco superior da crista ilíaca e a face externa da última costela. Consideraram-se com obesidade central os adolescentes com percentil ≥75, de acordo com o sexo e a faixa etária.^21^

A aferição da PAS e da PAD seguiu as recomendações 7^a^Diretriz Brasileira de Hipertensão Arterial,^13^ em sala de aula isolada e ambiente silencioso, utilizando-se aparelhos de pressão automáticos OMRON705-IT.^22^ Foram realizadas duas mensurações da PAS e da PAD no braço direito do avaliado, por enfermeiras voluntárias, com intervalo de cinco minutos entre elas, por enfermeiras voluntárias. Para a classificação de hipertensão, foram considerados os critérios de idade, sexo e percentil de estatura.^13^ Deste modo, valores abaixo do percentil 90 foram considerados adequados (normotensos), desde que inferiores a 120×80mmHg, crianças com percentis entre 90 e 95 foram enquadradas como pré-hipertensas (limítrofe) e com percentil igual ou superior a 95 foram consideradas hipertensas.

O nível de atividade física foi avaliado pelo IPAQ.^23^ As perguntas avaliadas referem-se às atividades físicas praticadas na semana anterior à aplicação do questionário. Classificaram-se os escolares como suficientemente ativos (ativos ou muito ativos) ou insuficientemente ativos (irregularmente ativos A e B ou sedentários).^23^

### Análise estatística

A análise estatística foi realizada pelo software *Statistical Package for the Social Science* (SPSS), versão 24. A normalidade dos dados foi aferida pelo teste Shapiro-Wilk. Para comparação entre sexos, estado nutricional, classificação da CC e AF-mv foram utilizados o teste *t-Student* independente para as variáveis paramétricas e o teste de Mann-Whitney para as não paramétricas. Utilizou-se o teste Qui-quadrado para avaliar as proporções entre os escolares considerados adequados, pré-hipertensos e hipertensos, de acordo com valores de PAS e PAD elevados. A análise de *odds ratio* foi usada para indivíduos considerados adequados e hipertensos, a partir das variáveis antropométricas e da AF-mv, por meio de regressão logística binária. Considerou-se o nível de significância de p<0,05 para todas as análises.

## Resultados

O excesso de peso foi encontrado em 35,11% dos 336 escolares avaliados, sendo 12,5% classificados como obesos. Foram classificados com obesidade central 13,39% dos escolares, 59,8% como suficientemente ativos em práticas de AF-mv e 52,97% apresentaram PA elevada, sendo 12,5% pré-hipertensos e 40,5% hipertensos. A distribuição da amostra de acordo com o sexo e a faixa etária está apresentada na Tabela 1 .

**Tabela 1:** – Distribuição da amostra por sexo e faixa etária

Faixa etária (anos)	Sexo masculino (n)	Sexo feminino (n)
12	33	29
13	38	29
14	34	36
15	23	36
**16**	**21**	**29**
**17**	**14**	**15**

Observou-se que os valores médios de PAS (p<0,001), IMC-z (p=0,034), IMC (p=0,001) e CC (p<0,001) foram maiores em meninos do que em meninas. Por outro lado, as meninas apresentaram valores superiores de PAD (p=0,009). Além disso, meninos participaram por mais tempo de atividades físicas leves a vigorosas do que meninas (p=0,007; p=0,009) ( Tabela 2 ).

**Tabela 2:** – Caraterísticas da amostra

	Total (n=336)	Sexo feminino (n=173)	Sexo masculino (n=163)	p
**Idade (anos)#**	14,50 [13,35 a 15,88]	14,74 [13,52 a 15,99]	14,35 [13,16 a 15,54]	0,103
**Peso corporal (kg)#**	56,25 [47,90 a 64,60]	54,70 [46,90 a 62,00]	58,90 [49,35 a 70,50]	0,001
**Estatura (cm)**	1,62 [0,10]	1,59 [0,07]	1,66 [0,10]	<0,001
**Estatura-z#**	0,49 [0,29 a 0,78]	0,44 [0,26 a 0,71]	0,57 [0,34 a 0,82]	0,003
**CC (cm)#**	68,00 [62,50 a 74,00]	65,00 [60,00 a 70,00]	71,00 [65,50 a 78,00]	<0,001
**IMC (kg/m** ^**2**^ **)#**	21,29 [19,00 a 24,09]	21,55 [19,17 a 24,01]	20,79 [18,87 a 24,80]	1,000
**IMC-z**	0,51 [1,22]	0,37 [1,16]	0,65 [1,27]	0,034
**PAS (mmHg)#**	124,00 [113,50 a 132,50]	120,00 [111,00 a 131,00]	127,00 [118,00 a 135,25]	<0,001
**PAD (mmHg)#**	69,50 [63,38 a 75,00]	70,00 [65,00 a 76,50]	68,00 [61,75 a 74,00]	0,009
**AF-L (min/dia)#**	19,29 [2,50 a 42,86]	14,29 [0,00 a 38,57]	25,71 [8,57 a 48,21]	0,007
**AF-Mod (min/dia)#**	17,14 [6,43 a 34,64]	12,86 [6,43 a 34,29]	17,14 [6,79 a 37,14]	0,282
**AF-Vig (min/dia)#**	7,14 [0,00 a 17,14]	5,71 [0,00 a 14,29]	8,57 [0,00 a 25,71]	0,009

*DP: desvio padrão; #não paramétricas; PAS: pressão arterial sistólica; PAD: pressão arterial diastólica; IMC: índice de massa corporal; CC: circunferência da cintura; IMC-z: IMC-escore Z; AF-L: atividade física leve; AF-Mod: atividade física moderada; AF-Vig: atividade física vigorosa.*

Na amostra total, identificou-se que os indivíduos com obesidade geral apresentaram maiores valores de PAS (p<0,001) e estatura-z (p=0,005) do que eutróficos ( Tabela 3 ). De acordo com o sexo, observou-se que tanto meninos quanto meninas com obesidade geral apresentaram maiores valores de PAS (p=0,003; p=0,001) e CC (p<0,001; p<0,001), entretanto somente as meninas apresentaram maiores valores de estatura-z e maior participação em atividades leves e vigorosas em comparação às eutróficas (p< 0,05).

**Tabela 3:** – Variáveis antropométricas e pressão arterial de acordo com a classificação do IMC-escore z (IMC-z) ♀

	EUTRÓFICO			EXCESSO DE PESO					

	Total (n=218)	Sexo feminino (n=119)	Sexo masculino (n=99)	Total (n=118)	Sexo feminino (n=54)	Sexo masculino (n=64)	pt	p ♀	p ♂
**Idade (anos)#**	14,66 [13,41 a 15,90]	14,80 [13,68 a 16,01]	14,49 [13,16 a 15,48]	14,25 [13,14 a 15,83]	14,62 [13,14 a 15,86]	14,07 [13,21 a 15,61]	0,232	0,356	0,573
**Peso corporal (kg)#**	51,60 [43,92 a 57,77]	51,10 [43,30 a 55,90]	52,20 [45,65 a 59,15]	67,45 [61,12 a 78,62]	64,00 [59,70 a 71,15]	74,10 [64,75 a 83,15]	<0,001	<0,001	<0,001
**Estatura (cm)**	1,61 [0,10]	1,58 [0,08]	1,65 [0,11]	1,63 [0,09]	1,60 [0,06]	1,66 [0,10]	0,062	0,230	0,380
**Estatura-z#**	0,43 [0,27 a 0,73]	0,41 [0,23 a 0,64]	0,54 [0,32 a 0,79]	0,57 [0,35 a 0,83]	0,52 [0,35 a 0,74]	0,71 [0,42 a 0,87]	0,005	0,027	0,083
**CC (cm)#**	64,50 [60,00 a 69,00]	61,00 [58,00 a 66,00]	66,50 [63,75 a 70,20]	76,50 [72,00 a 86,00]	72,50 [67,25 a 76,50]	83,00 [75,25 a 91,12]	<0,001	<0,001	<0,001
**IMC (kg/m** ^**2**^ **)#**	19,54 [18,07 a 21,26]	19,69 [18,18 a 21,65]	19,33 [17,81 a 20,49]	25,34 [23,88 a 27,91]	24,93 [24,08 a 27,08]	26,04 [23,64 a 29,03]	<0,001	<0,001	<0,001
**IMC-z**	-0,18 [0,85]	-0,19 [0,90]	-0,17 [0,78]	1,78 [0,64]	1,60 [0,56]	1,93 [0,68]	<0,001	<0,001	<0,001
**PAS (mmHg)#**	121,00 [111,50 a 130,50]	117,00 [108,50 a 129,00]	124,00 [116,50 a 132,00]	129,00 [120,00 a 136,50]	124,00 [116,62 a 136,25]	130,25 [122,38 a 138,75]	<0,001	0,001	0,003
**PAD (mmHg)#**	68,50 [62,50 a 74,00]	70,00 [64,50 a 75,25]	67,50 [61,25 a 73,25]	70,00 [64,50 a 76,50]	70,50 [66,12 a 77,88]	69,50 [62,38 a 74,62]	0,075	0,255	0,096
**AF-L (min/dia)#**	17,14 [0,00 a 42,32]	11,43 [0,00 a 36,43]	25,71 [8,57 a 51,43]	24,29 [6,96 a 51,43]	24,29 [1,43 a 64,29]	23,57 [8,57 a 43,39]	0,090	0,042	0,910
**AF-Mod (min/dia)#**	14,29 [6,43 a 34,29]	12,86 [6,07 a 31,07]	17,14 [6,79 a 37,14]	17,14 [8,57 a 35,36]	13,57 [8,57 a 35,36]	19,29 [8,04 a 35,36]	0,532	0,768	0,734
**AF-Vig (min/dia)#**	5,71 [0,00 a 17,14]	4,29 [0,00 a 13,57]	8,57 [0,00 a 29,29]	8,57 [2,14 a 17,14]	8,57 [3,21 a 17,14]	10,71 [1,39 a 17,14]	0,120	0,010	0,603

*DP = desvio padrão; #não paramétricas; t: Total vs Total; ♀: meninas versus meninas; ♂ : meninos versus meninos; PAS: pressão arterial sistólica; PAD: pressão arterial diastólica; IMC: índice de massa corporal; CC: circunferência da cintura; AF-L: atividade física leve; AF-Mod: atividade física moderada; AF-Vig: atividade física vigorosa.*

No grupo com obesidade central, as meninas apresentaram maiores valores de PAS (p=0,026), peso corporal (p<0,001), estatura (p=0,038) e IMC-z (p<0,001) em comparação ao grupo considerado adequado. Em relação aos meninos, o grupo com obesidade central apresentou maiores valores de PAS (p=0,002), PAD (p=0,003), IMC (p<0,001) e IMC-z (p<0,001) em comparação ao grupo adequado ( Tabela 4 ). Entretanto, não foram identificadas diferenças para a prática de atividade física.

**Tabela 4:** – Variáveis antropométricas e pressão arterial de acordo com a classificação da circunferência da cintura (CC)

	ADEQUADO			OBESIDADE CENTRAL					

	Total (n=291)	Sexo feminino (n=163)	Sexo masculino (n=128)	Total (n=45)	Sexo feminino (n=10)	Sexo masculino (n=35)	pt	p ♀	p ♂
**Idade (anos)#**	14,64 [13,39 a 15,94]	14,74 [13,64 a 16,01]	14,49 [13,23 a 15,63]	13,81 [12,99 a 15,52]	13,50 [12,95 a 15,48]	13,94 [13,02 a 15,31]	0,065	0,170	0,326
**Peso corporal (kg)#**	54,50 [46,80 a 61,35]	53,80 [46,45 a 60,80]	55,20 [47,58 a 61,62]	78,40 [68,90 a 89,30]	81,75 [70,65 a 89,38]	78,40 [68,80 a 88,20]	**<0,001**	**<0,001**	**<0,001**
**Estatura (cm)**	1,62 [0,10]	1,59 [0,07]	1,65 [0,11]	1,66 [0,08]	1,64 [0,03]	1,67 [0,09]	**0,005**	**0,038**	0,543
**Estatura-z#**	0,45 [0,28 a 0,74]	0,43 [0,24 a 0,68]	0,55 [0,32 a 0,80]	0,74 [0,49 a 0,89]	0,77 [0,61 a 0,90]	0,73 [0,46 a 0,89]	**<0,001**	**0,005**	0,064
**CC (cm)#**	66,00 [61,00 a 71,50]	64,00 [59,00 a 69,50]	69,50 [64,50 a 72,50]	88,50 [84,00 a 95,00]	81,00 [79,00 a 88,50]	90,00 [86,00 a 97,25]	**<0,001**	**<0,001**	**<0,001**
**IMC (kg/m2)#**	20,56 [18,54 a 22,77]	21,40 [19,13 a 23,38]	20,11 [18,37 a 21,60]	28,67 [26,40 a 31,40]	29,80 [26,30 a 33,16]	28,67 [26,66 a 30,23]	**<0,001**	**<0,001**	**<0,001**
**IMC-z**	0,22 [1,02]	0,25 [1,07]	0,17 [0,95]	2,39 [0,52]	2,35 [0,53]	2,40 [0,53]	**<0,001**	**<0,001**	**<0,001**
**PAS (mmHg)#**	122,50 [112,75 a 131,00]	119,00[111,00 a 129,75]	124,25[117,50 a 132,88]	132,00[127,00 a 139,50]	136,25[129,00 a 138,25]	131,00[126,75 a 141,75]	**<0,001**	**0,026**	**0,002**
**PAD (mmHg)#**	68,50 [62,50 a 74,50]	70,00[65,00 a 75,75]	67,00[61,00 a 73,50]	73,00[68,00 a 78,00]	76,50[68,88 a 84,38]	72,50[68,25 a 74,75]	**0,009**	0,101	**0,003**
**AF-leve (min/dia)#**	19,29 [0,00 a 42,86]	14,29[0,00 a 38,57]	25,71[8,57 a 46,61]	21,43[5,71 a 51,43]	23,57[9,29 a 57,86]	21,43[5,00 a 47,14]	0,609	0,432	0,527
**AF-moderada (min/dia) #**	14,29 [6,43 a 35,00]	12,86[6,43 a 33,21]	17,14[6,43 a 36,43]	17,14[8,57 a 34,29]	19,29[6,96 a 33,75]	17,14[9,64 a 34,29]	0,377	0,779	0,619
**AF-vigorosa (min/dia)#**	6,43 [0,00 a 17,14]	5,71[0,00 a 14,29]	8,57[0,00 a 26,43]	10,71[2,86 a 17,14]	6,43[0,71 a 11,79]	12,86[3,57 a 22,14]	0,271	0,928	0,607

*#não paramétricas; t: total versus total; ♀: meninas versus meninas; ♂: meninos versus meninos; PAS: pressão arterial sistólica; PAD: pressão arterial diastólica; IMC: índice de massa corporalIMC-z = IMC-escore z.*

As medidas antropométricas e de PA também foram avaliadas de acordo com a AF-mv ( Tabela 5 ). Na amostra total, os indivíduos ativos apresentaram maiores valores de peso corporal (p=0,002), IMC-z (p=0,016), estatura-z (p=0,001), além da PAD mais baixa (p=0,046) em relação aos classificados como insuficientemente ativos. Em relação às meninas consideradas suficientemente ativas, foram identificados maiores valores para IMC (p=0,005), estatura (p=0,048), CC (p=0,015), IMC-z (p=0,005) e estatura-z (p=0,016) em relação às insuficientemente ativas. Os meninos suficientemente ativos possuíam estatura-z maior (p=0,025) em relação aos insuficientemente ativos.

**Tabela 5:** – Variáveis antropométricas e pressão arterial de acordo com a classificação de nível de atividade física, por sexo

	SUFICIENTEMENTE ATIVO			INSUFICIENTEMENTE ATIVO					

	Total (n=201)	Sexo feminino (n=93)	Sexo masculino (n=108)	Total (n=135)	Sexo feminino (n=80)	Sexo masculino (n=55)	pt	p ♀	p ♂
**Idade (anos)#**	14,39[13,17-15,96]	14,48[13,28-15,99]	14,27[13,08-15,88]	14,64[13,56-15,80]	14,84[13,94-15,95]	14,45[13,38-15,10]	0,504	0,442	0,854
**Peso corporal (kg)#**	58,10[50,50-65,80]	57,40[50,70-63,40]	59,15[50,40-72,30]	53,70[43,90-62,05]	51,60[43,08-60,83]	58,50[48,45- 66,65]	0,002	0,005	0,300
**Estatura (cm)**	1,63[0,10]	1,60[0,07]	1,67[0,11]	1,60[0,09]	1,58[0,08]	1,64[0,10]	0,002	0,048	0,114
**Estatura-z#**	0,56[0,34-0,80]	0,48[0,34-0,73]	0,68[0,35- 0,84]	0,41[0,23- 0,70]	0,38[0,22-0,62]	0,48[0,27-0,71]	0,001	0,016	0,025
**CC (cm)#**	69,50[64,00-75,00]	67,00[61,00-72,00]	71,25[66,38-79,25]	65,00[61,00-72,00]	63,00[59,00-67,00]	70,00[64,00-75,50]	0,001	0,015	0,226
**IMC (kg/m** ^**2**^ **)#**	21,58[19,40-24,50]	22,27[19,69-24,26]	20,98[19,17-24,80]	20,35[18,23-23,56]	20,10[18,25-23,34]	20,39[18,29-24,62]	0,020	0,009	0,530
**IMC**_**-z**_	0,64[1,11]	0,59[1,01]	0,67[1,19]	0,31[1,34]	0,11[1,26]	0,61[1,41]	0,016	0,005	0,748
**PAS (mmHg)#**	124,50[115,50-132,50]	120,50[112,50-131,00]	129,00[119,12-136,62]	123,00[111,50-130,75]	117,50[108,50-130,62]	124,00[118,00-130,75]	0,067	0,365	0,205
**PAD (mmHg)#**	69,00[62,50-74,00]	70,50[64,50-75,00]	67,75[61,38-73,00]	70,00[64,50-77,75]	70,00[65,00-77,62]	70,00[62,50-77,75]	0,046	0,599	0,063
**AF-L (min/dia)#**	34,29[12,86-64,29]	34,29[14,29-64,29]	34,29[12,86-64,29]	8,57[0,00-17,14]	0,00[0,00-12,86]	12,86[2,86-25,71]	<0,001	<0,001	<0,001
**AF-Mod (min/dia)#**	25,71[12,86-60,00]	25,71[12,86-60,00]	25,71[12,86-53,57]	8,57[0,00-12,93]	8,57[0,00-12,86]	8,57[0,71-17,14]	<0,001	<0,001	<0,001
**AF-Vig (min/dia)#**	14,29[5,71-32,14]	12,86[5,71-25,71]	15,71[8,04-39,64]	0,00[0,00-5,71]	0,00[0,00-4,64]	1,86[0,00-5,71]	<0,001	<0,001	<0,001

*#não paramétricas; t: total versus total; ♀: meninas versus meninas; ♂: meninos versus meninos; PAS: pressão arterial sistólica; PAD: pressão arterial diastólica; IMC: índice de massa corporal; CC: circunferência da cintura; IMC-z: IMC-escore Z; AF-L: atividade física leve; AF-Mod: atividade física moderada; AF-Vig: atividade física vigorosa.*

Analisando-se os parâmetros antropométricos CC, IMC-z e AF-mv com a PA ( Tabela 6 ), os meninos apresentaram maior proporção de pré- hipertensão e hipertensão arterial (p=0,033). Os indivíduos considerados ativos apresentaram maior proporção de pré-hipertensão diastólica, e os sedentários apresentaram maior proporção de HA (p=0,015). Constatou-se que os adolescentes com obesidade central e aqueles com excesso de peso apresentaram maior proporção de pré-hipertensão e hipertensão e PAS elevada (p<0,001).

**Tabela 6:** – Distribuição percentual de pressão arterial de acordo com valores antropométricos e nível de atividade física

		PRESSÃO ARTERIAL			p	PRESSÃO ARTERIAL SISTÓLICA			p	PRESSÃO ARTERIAL DIASTÓLICA			p

		Adequado (n=158)	Pré (n=42)	Hipertenso (n=136)		Adequado (n=164)	Pré (n=40)	Hipertenso (n=132)		Adequado (n=287)	Pré (n=18)	Hipertenso (n=31)	
**Sexo (%)**	Feminino	91 (52,6%)	15 (8,7%)	67 (38,7%)	0,033	93 (53,8%)	15 (8,7%)	65 (37,6%)	0,075	145 (83,8%)	11 (6,4%)	17 (9,8%)	0,633
	Masculino	67 (41,1%)	27 (16,6%)	69 (42,3%)		71 (43,6%)	25 (15,3%)	67 (41,1%)		142 (87,1%)	7 (4,3%)	14 (8,6%)	
**AF-mv (%)**	Suf. ativo	91 (45,3%)	27 (13,4%)	83 (41,3%)	0,685	93 (46,3%)	27 (13,4%)	81 (40,3%)	0,412	179 (89,1%)	11 (5,5%)	11 (5,5%)	0,015
	Insuf. ativo	67 (49,6%)	15 (11,1%)	53 (39,3%)		71 (52,6%)	13 (9,6%)	51 (37,8%)		108 (80,0%)	7 (5,2%)	20 (14,8%)	
**CC (%)**	Adequado	151 (51,9%)	34 (11,7%)	106 (36,4%)	<0,001	155 (53,3%)	32 (11,0%)	104 (35,7%)	<0,001	252 (86,6%)	16 (5,5%)	23 (7,9%)	0,103
	Obes. central	7 (15,6%)	8 (17,8%)	30 (66,7%)		9 (20,0%)	8 (17,8%)	28 (62,2%)		35 (77,8%)	2 (4,4%)	8 (17,8%)	
**IMC-z (%)**	Eutrofia	121 (55,5%)	25 (11,5%)	72 (33,0%)	<0,001	125 (57,3%)	22 (10,1%)	71 (32,6%)	<0,001	190 (87,2%)	13 (6,0%)	15 (6,9%)	0,114
	Excesso de peso	37 (31,4%)	17 (14,4%)	64 (54,2%)		39 (33,1%)	18 (15,3%)	61 (51,7%)		97 (82,2%)	5 (4,2%)	16 (13,6%)	

*AF-mv: atividade física moderada-vigorosa; Insuf.: insuficientemente; Suf.: suficientemente; Obes.: obesidade; CC: circunferência da cintura; IMC-z: índice de massa corporal escore z.*

Segundo a análise de risco de chance ( Tabela 7 ), não houve diferença entre os sexos para HA (OR=1,40; IC 0,88 a 2,22), PAS (OR=1,35; IC 0,85 a 2,14) e PAD (OR=0,84; IC 0,40 a 1,77) elevadas. Por outro lado, de acordo com os indicadores de adiposidade, os indivíduos com obesidade central apresentaram 6,11 (IC 2,59 a 14,42) vezes mais chances de ter HA em relação àqueles com CC adequadas, e os indivíduos com obesidade geral apresentaram 2,91 (IC=1,77=4,79) vezes mais em relação aos que apresentaram IMC-escore z adequado. Além disso, foi observado que os adolescentes suficientemente ativos apresentaram redução de aproximadamente 1/3 do risco de PAD elevada (OR=0,33; IC95%: 0,15 a 0,72).

**Tabela 7:** – *Odds ratios* (OR) para o risco de pressão arterial elevada entre as variáveis antropométricas e o nível de atividade física

	HA OR (IC95%)	PAS elevada OR (IC95%)	PAD elevada OR (IC95%)
**Sexo masculino**	1,40 (0,88 a 2,22)	1,35 (0,85 a 2,14)	0,84 (0,40 a 1,77)
**AF-mv (suficientemente ativo)**	1,15 (0,72 a 1,84)	1,21 (0,76 a 1,93)	0,33 (0,15 a 0,72)
**CC (obesidade central)**	6,11 (2,59 a 14,42)	4,64 (2,10 a 10,23)	2,50 (1,04 a 6,03)
**IMC-z (Obesidade geral)**	2,91 (1,76 a 4,79)	2,75 (1,68 a 4,52)	2,09 (0,99 a 4,40)

*AF-mv: atividade física moderada-vigorosa; CC: circunferência da cintura; IMC-z: índice de massa corporal escore z; há: hipertensão arterial; PAS: pressão arterial sistólica; PAD: pressão arterial diastólica; IC: intervalo de confiança; valores de p com nível de significância p<0,005.*

## Discussão

Os principais resultados revelaram maior risco para HA em escolares com obesidade abdominal (OR=6,11) e obesidade geral (OR=2,91). Além disso, adolescentes que praticam AF-mv apresentaram redução de 33% do risco de PAD elevada. A literatura atual tem sido consistente em demonstrar que o IMC-z e a CC estão fortemente associados com HA na infância e na adolescência.^9^ Em acréscimo, os achados deste estudo exibem relevante fator protetivo da prática de AF-mv para a HA na adolescência, aspecto que foi pouco explorado em estudos populacionais.

De acordo com todo o exposto, as medidas antropométricas representam significativos preditores de HA, que se justificam como alternativa simples, rápida, de fácil interpretação e de bom custo-efetividade.^24 , 25^ Vários relatos demonstram associação entre PA, IMC e CC, sugerindo a obesidade como forte fator de risco para o desenvolvimento de HA na vida adulta.^9 , 16^ A distribuição excessiva de gordura visceral é acompanhada por alterações de vários marcadores inflamatórios e endoteliais^26^ que estimulam o aumento de eventos de resistência à insulina, a disfunção endotelial e a elevação da retenção de líquido, que podem promover variações nos níveis de PA e crescimento do risco cardiovascular.^27^

Identificou-se que 40,5% dos adolescentes apresentaram HA, sendo metade dos escolares com excesso de peso e 2/3 com CC elevada foram diagnosticados como HA em maior proporção em relação a adolescentes com medidas adequadas. Em estudo de representatividade nacional e regional que avaliou 73.399 estudantes com idades entre 12 e 17 anos da região Sul do Brasil, estimou-se que a prevalência de HA foi de 12,5% e de pré-hipertensão foi de 17%, o excesso de peso variou entre 29,8%^1^ e 35,5%^28^ dos adolescentes sul-brasileiros. Sugere-se que, além dos fatores genéticos e ambientais, a obesidade e a HA podem estar relacionadas às disfunções metabólicas.^27^ Com relação às diferenças entre sexos, foram identificadas prevalência de HA e PAS similares entre meninos e meninas, entretanto, as meninas apresentaram maiores médias de PAD. Resultados semelhantes foram encontrados na literatura.^9^ Uma possível explicação deve-se ao fato de que as meninas praticam menos atividades físicas por dia em relação aos meninos, o que demonstrou efeito protetivo para PAD elevada.

Além disso, observou-se que meninas com excesso de peso praticam maior tempo de atividades físicas, bem como adolescentes considerados ativos apresentaram maiores médias de indicadores antropométricos. Este dado pode refletir a participação em atividades físicas como uma estratégia para reduzir o peso corporal.^14^

Identificou-se que altos valores de CC e IMC-z foram associados ao maior risco de HA, entretanto, os considerados suficientemente ativos apresentaram redução de 1/3 do risco de PAD elevada, o que sugere que AF-mv pode interferir nos níveis pressóricos, além de reduzir o risco metabólico.^29^ Entretanto, outro estudo^30^ demonstrou que somente o sobrepeso e a obesidade associaram-se diretamente com HA, mas não a prática de AF-mv.^31^ Grande parte dos adolescentes foram considerados suficientemente ativos, o que pode estar relacionado ao nível socioeconômico, com a oferta de atividades físicas fora dos períodos escolares.^32^

O processo de urbanização, os avanços tecnológicos da sociedade moderna e o aumento da violência estão associados com a mudança no comportamento de crianças e adolescentes.^33^ O aumento do tempo em atividades sedentárias e a menor prática AF-mv favorecem o ganho de peso e as doenças associadas à obesidade, entre elas, a HA.^1^ Recomenda-se a prática de no mínimo de 300 minutos de AF-mv por semana para fornecer benefícios adicionais de saúde.^34^

Neste sentido, o aspecto de relevância encontrado neste estudo refere-se à associação entre valores mais baixos de PAD em adolescentes que praticam AF-mv, sugerindo que essa prática possa interferir nos níveis pressóricos na população infantojuvenil.^30^ Estudo recente mostrou que adolescentes com melhores aptidões musculares exibiram menores níveis de PAD.^35^ Por todos os motivos expostos, conclui-se que intervenções que estimulem a transição da inatividade física para um estágio de ação promovem impactos imediatos para o aumento da prática da atividade física em escolares,^36^ o que pode ser considerado fator de proteção para HA.

Portanto, a eficácia em detectar precocemente fatores de risco pode contribuir para prevenção de doenças cardiovasculares na vida adulta, apesar de mudanças de hábitos e atitudes representarem tarefas complexas e muitas vezes de resultados insatisfatórios. Entretanto, políticas de saúde direcionadas para escolares, assim como investimentos sociais para melhoria da prática de AF-mv, poderão no futuro determinar mudanças significativas da população. Neste sentido, é de grande importância que profissionais da educação e da saúde promovam o controle e a prevenção da HA, assim como de outros fatores de risco associados a doenças cardiovasculares.

Este estudo apresenta algumas limitações como o pequeno número da amostra e a quantidade de vezes que a PA foi aferida (deve ser realizada em pelo menos três ocasiões distintas para melhor diagnosticar os escolares hipertensos). Outra limitação é o uso de questionário recordatório para avaliar o nível de atividade física, porém o IPAQ apresenta excelente associação com AF-mv.^23^ Também não foram verificadas as variáveis socioeconômicas, maturação sexual, ingestão de sal da dieta e antecedentes familiares para HA. Entretanto, vale ressaltar que o ponto forte foi associar a HA ao diagnóstico de obesidade central e obesidade geral, bem como destacar a importância da prática de AF-mv como fator de proteção às alterações na PA em crianças e adolescentes. Tais avaliações são importantes como forma preventiva em saúde pública, pois muitas crianças e adolescentes não apresentam a oportunidade de terem a PA avaliada na escola.

## Conclusões

Neste estudo, observou-se que metade dos escolares avaliados apresentaram HA e 1/3 obesidade geral. Além disso, as medidas antropométricas de CC e IMC-z foram significativamente relacionadas ao maior risco de HA, e a prática de atividades físicas aparece como fator preventivo de PAD elevada em crianças e adolescentes. Desta forma, sugere-se a implantação de programas que estimulem o estilo de vida saudável no ambiente escolar, para contribuir com a redução dos indicadores de obesidade central e obesidade geral, bem como prevenir contra HA ao aumentar a prática de AF-mv na população infantojuvenil.
