# Supplementary material for: Medidas Hipertensivas em Escolares: Risco da Obesidade Central e Efeito Protetor da Atividade Física Moderada-Vigorosa
Source: Arq Bras Cardiol. 2020 Jul 28;115(1):42–9. [Article in Portuguese] doi: 10.36660/abc.20180391 (PMC8384320; doi:10.36660/abc.20180391)
Supplement: Translation

# Translation: Hypertensive Measures In Schoolchildren: Risk Of Central Obesity And Protective Effect Of Moderate-To-Vigorous Physical Activity

**Authors:** Tatiana Affornali Tozo, Beatriz Oliveira Pereira, Francisco José de Menezes, Cristianne Morgado Montenegro, Carla Marisa Maia Moreira, Neiva Leite

**Affiliations:** 1 Portugal Universidade do Minho, Braga - Portugal; 2 Brazil Universidade Federal do Paraná, Curitiba, PR - Brazil; 3 Portugal Universidade do Porto, Porto - Portugal

**Keywords:** Child, Adolescent, School Children, Physical Activity, Waist Circumference, Body Mass Index, Hipertension, Blood Pressure

## Abstract

**Background:**

The proportion of arterial hypertension (AH) has increased in children and adolescents and is associated with several comorbidities.

**Objective:**

To verify the association of arterial hypertension with central and general obesity as well as according to the level of physical activity in schoolchildren.

**Methods:**

336 children and adolescents aged 11 to 17 participated in the study. Height, body weight, waist circumference (WC) and blood pressure (BP) were measured. The body mass index z-score (BMI-z) was calculated. The level of physical activity was assessed by the short form of the International Physical Activity Questionnaire (IPAQ) according to the practice of moderate-to-vigorous physical activities (AF-mv). Students with systolic (SBP) and/or diastolic blood pressure (DBP) higher than the 95^th^percentile according to sex, age and height or ≥120/80 were considered hypertensive. Statistical tests of t-Student, Chi-square, Mann-Whitney and binary logistic regression model were used, considering the significance level of p<0.05.

**Results:**

It was found that 40.5% of the students had AH, 35.11% were overweight (12.5% obese), 13.39% had high WC and 40.2% were considered insufficiently active in AF-mv. The chances of AH were related to high WC (OR = 6.11; 95% CI: 2.59¬–14.42) and overweight (OR = 2.91; 95% CI: 1.76–4.79). In addition, adolescents who practiced AF-mv had a lower risk of high DBP (OR = 0.33; 95% CI: 0.15–0.72).

**Conclusion:**

Central obesity was the best predictor of AH in children and adolescents, as well as general obesity and males. The practice of AF-mv demonstrated a protective effect on high DBP in schoolchildren. (Arq Bras Cardiol. 2020; 115(1):42-49)

## Introduction

The frequency of arterial hypertension (AH) has increased in all age groups and in several countries,^1^ affecting children and adolescents, and tends to persist over time, with a high probability of progressing into adulthood,^2^ mainly due to the increasing prevalence of obesity,^3^ which is associated with the appearance of several comorbidities.^4^

The joint analysis of lifestyle habits that may predispose to the onset of cardiovascular diseases in adulthood plays an important role in preventing hypertension in children and adolescents.^5^ Obesity has a multifactorial origin, involving aspects of behavior related to diet, physical activity and psychological factors.^6^

Therefore, early diagnosis of AH in children and adolescents is relevant to prevent the disease from advancing into adulthood, reduce the risk of cardiovascular problems^7^ and recommend therapeutic programs to stop the process.^8^ Thus, anthropometric measures such as body mass index (BMI) and waist circumference (WC) are efficient low-cost indicators for identifying cardiovascular risks.^9^ BMI classified as overweight demonstrates general obesity,^10^ while the largest WC is related to central obesity,^11^ the latter being more associated with inflammation in adults and appearance of cardiometabolic comorbidities.^12^

Blood pressure should be measured on three different occasions^13^ to confirm the diagnosis of AH, whereas in epidemiological studies it is usually measured on one day and the term hypertensive measures has been used.^14^ Therefore, in children and adolescents, some studies show greater associations between AH with central obesity^15^ and with general obesity,^16^ which generates controversy as to the location of adiposity and blood pressure (BP) in this population.

In addition, evidence on the practice of physical activities with moderate-to-vigorous intensity (AF-mv) and BP is still limited, as well as the relationship between anthropometric measures and AF-mv as protectors of AH in children and adolescents. Thus, it is important to identify the risk of AH in adolescence to prevent the advancement of this condition in adulthood, which can increase the efficiency of treatment. Therefore, the present study aims to verify the association between AF-mv and anthropometric indicators of obesity with the diagnosis of AH in children and adolescents.

## Methods

This is a descriptive cross-sectional quantitative study carried out in the city of São José dos Pinhais, Paraná (southern region of Brazil). The sample consisted of conglomerates, chosen for convenience, in which each private elementary and high school institution in the city was considered a conglomerate. Of the six institutions located in the central region that were invited, only two private schools agreed to participate in the study, to which all elementary and high school students were invited.

In the city, approximately 55,289 students were attending the final grades of elementary school and high school in 2018.^17^ The prevalence of 12.5% of hypertensive children and adolescents in the southern region of Brazil was stipulated.^18^ Based on the probabilistic sample selection, the total number of 111 adolescents for inference of the student population in the stipulated age range was obtained. 1.5x subjects were included regarding the design effect, taking into account a 5% sample error, and an additional 30% were included for possible dropouts, resulting in a total of 217 individuals aged between 11 and 17.

The study included 336 volunteer children and adolescents aged 11 to 17, of both sexes (173 girls). Pregnant women, individuals with limitations that prevented them from participating in any study procedure, and those who did not have signed the Free and Informed Consent Term (FICT) and the Free and Informed Consent Term for minors of age (TALE) were excluded from the study. All procedures were approved by the Research Ethics Committee of Pontifícia Universidade Católica do Paraná, PUC – PR, CAAE (71324017.1.0000.0020/2017).

Anthropometric measurements were collected at school, in a standardized manner, following the procedures recommended by the Anthropometric Standardization Reference Manual.^19^ Height was measured using a portable stadiometer, with a resolution of 0.1 centimeters (cm); height was expressed in cm. Body weight was assessed with a portable scale model PLENA, with resolution of up to 100 grams and capacity of 150 kg.

BMI z-score (BMI-z)^20^ was calculated using WHO Anthro Plus® version 1.0.4. Participants with BMI-z between ≥ -2 and <+1 were classified as overweight; between ≥ 1 and <2, obese; those with ≥2 were classified as eutrophic according to age and sex. Adolescents classified as overweight and obese (BMI-z ≥1) were considered with general obesity. In order to measure WC, an inelastic measuring tape was used at the midpoint between the last upper arch of the iliac crest and the outer face of the last rib. Adolescents with a ≥75 percentile were considered with central obesity according to sex and age group.^21^

Measurement of systolic blood pressure (SBP) and diastolic blood pressure (DBP) followed the recommendations of the 7^th^Brazilian Guideline on Hypertension^13^ and were collected in a quiet isolated classroom using the automatic pressure device OMRON705-IT.^22^ Two measurements of SBP and DBP were performed on the subject’s right arm by volunteer nurses with an interval of five minutes between them. These measurements were classified according to age, sex and height percentile.^13^ Thus, the criteria for classification were: values below the 90^th^percentile were considered adequate (normotensive), since they were lower than 120/80 mmHg; the percentiles between 90 and 95 were considered as prehypertensive (borderline); and equal to or greater than the 95^th^percentile were considered hypertensive.

The level of physical activity was assessed by the International Physical Activity Questionnaire — short form — (IPAQ).^23^ The questions refer to the physical activities practiced in the week prior to the application of the questionnaire. The individuals were classified as sufficiently active (active or very active) or insufficiently active (irregularly active A, B or sedentary).^23^

### Statistical analysis

Statistical analysis was performed using the Statistical Package for the Social Science (SPSS), v. 24. The normality of the data was assessed by the Shapiro Wilk test. For comparison between sexes, nutritional status, WC classification and AF-mv, the independent Student’s t test was used for parametric variables, and the Mann-Witney test for non-parametric variables. The Chi-square test was used to assess the proportions among students considered adequate, pre-hypertensive, hypertensive according to high SBP and DBP. The analysis of odd ratios for individuals considered adequate and hypertensive, based on anthropometric variables and AF-mv through binary logistic regression. The significance level of p<0.05 was considered for all analyses.

## Results

Excess weight was found in 35.11% of the 336 schoolchildren evaluated, with 12.5% being classified as obese. Central obesity was present in 13.39% of the students; 59.8% were classified as sufficiently active in PA-mv practices and 52.97% had high BP; 12.5% were pre-hypertensive and 40.5% hypertensive. The sample distribution according to sex and age group is shown in table 1 .

**Table 1:** – Sample distribution by sex and age range

Age range (years)	Male (n)	Female (n)
12	33	29
13	38	29
14	34	36
15	23	36
**16**	**21**	**29**
**17**	**14**	**15**

It was observed that mean SBP (p <0.001), BMI-Z (p = 0.034), body mass (p=0.001) and WC (p<0.001) were higher in boys than in girls. On the other hand, girls showed higher PAD (p=0.009). In addition, boys participated for longer in light and vigorous physical activities than girls (p=0.007; p=0.009) ( Table 2 ).

**Table 2:** – Sample characteristics

	Total (n=336)	Female (n=173)	Male (n=163)	W
**Age (years)#**	14.50[13.35–15.88]	14.74[13.52–15.99]	14.35[13.16-15.54]	0.103
**Body weight (kg)#**	56.25[47.90-64.60]	54.70[46.90-62.00]	58.90[49.35-70.50]	0.001
**Stature (cm)**	1.62[0.10]	1.59[0.07]	1.66[0.10]	<0.001
**Stature -z#**	0.49[0.29-0.78]	0.44[0.26-0.71]	0.57[0.34-0.82]	0.003
**WC (cm) #**	68.00[62.50-74.00]	65.00[60.00-70.00]	71.00[65.50-78.00]	<0.001
**BMI (kg/m** ^**2**^ **)#**	21.29[19.00-24.09]	21.55[19.17- 24.01]	20.79 [18.87. 24.80]	1.000
**BMI-Z**	0.51[1.22]	0.37[1.16]	0.65[1.27]	0.034
**SBP (mmHg)#**	124.00[113.50-132.50]	120.00[111.00-131.00]	127.00[118.00-135.25]	<0.001
**DBP (mmHg)#**	69.50[63.38-75.00]	70.00[65.00-76.50]	68.00[61.75-74.00]	0.009
**PA-light (min/day)#**	19.29[2.50-42.86]	14.29[0.00-38.57]	25.71[8.57-48.21]	0.007
**PA- Moderate (min/day)#**	17.14[6.43-34.64]	12.86[6.43-34.29]	17.14[6.79-37.14]	0.282
**PA- Vigorous (min/day)#**	7.14[0.00-17.14]	5.71[0.00-14.29]	8.57[0.00-25.71]	0.009

*SD: standard deviation; # non-parametric; SBP: systolic blood pressure; DBP: diastolic blood pressure; BMI; body mass index; WC; waist circumference; BMI-Z: BMI z-score; PA: physical activity.*

In the total sample, it was identified that individuals with general obesity had higher SBP (p<0.001) and height-z (p=0.005) than eutrophic individuals ( Table 3 ). According to sex, it was observed that both boys and girls with general obesity had higher SBP (p=0.003; p=0.001) and WC (p<0.001; p<0.001). However, only girls had higher values of height -z and greater participation in light and vigorous activities compared to eutrophic ones (p<0.05).

**Table 3:** – Anthropometric variables and blood pressure according to the classification of bmi z-score.

	EUTHROPHIC			OVERWEIGHT					

	Total (n=218)	Female (n=119)	Male (n=99)	Total (n=118)	Female (n=54)	Male (n=64)	pt	p ♀	p ♂
**Age (years)#**	14.66[13.41-15.90]	14.80[13.68-16.01]	14.49[13.16-15.48]	14.25[13.14-15.83]	14.62[13.14-15.86]	14.07[13.21-15.61]	0.232	0.356	0.573
**Body weight (kg)#**	51.60[43.92-57.77]	51.10[43.30-55.90]	52.20[45.65-59.15]	67.45[61.12-78.62]	64.00[59.70-71.15]	74.10[64.75-83.15]	<0.001	<0.001	<0.001
**Stature (cm)**	1.61[0.10]	1.58[0.08]	1.65[0.11]	1.63[0.09]	1.60[0.06]	1.66[0.10]	0.062	0.230	0.380
**Stature z#**	0.43[0.27- 0.73]	0.41[0.23-0.64]	0.54[0.32- 0.79]	0.57[0.35- 0.83]	0.52[0.35-0.74]	0.71[0.42-0.87]	0.005	0.027	0.083
**WC (cm)#**	64.50[60.00-69.00]	61.00[58.00-66.00]	66.50[63.75-70.20]	76.50[72.00-86.00]	72.50[67.25-76.50]	83.00[75.25-91.12]	<0.001	<0.001	<0.001
**BMI (kg/m** ^**2**^ **)#**	19.54[18.07-21.26]	19.69[18.18-21.65]	19.33[17.81-20.49]	25.34[23.88-27.91]	24.93[24.08-27.08]	26.04[23.64-29.03]	<0.001	<0.001	<0.001
**BMI- Z**	-0.18[0.85]	-0.19[0.90]	-0.17[0.78]	1.78[0.64]	1.60[0.56]	1.93[0.68]	<0.001	<0.001	<0.001
**SBP (mmHg)#**	121.00[111.50-130.50]	117.00[108.50-129.00]	124.00[116.50-132.00]	129.00[120.00-136.50]	124.00[116.62-136.25]	130.25[122.38-138.75]	<0.001	0.001	0.003
**DBP (mmHg)#**	68.50[62.50-74.00]	70.00[64.50-75.25]	67.50[61.25-73.25]	70.00[64.50-76.50]	70.50[66.12-77.88]	69.50[62.38-74.62]	0.075	0.255	0.096
**PA-L (min/day) #**	17.14[0.00-42.32]	11.43[0.00-36.43]	25.71[8.57-51.43]	24.29[6.96-51.43]	24.29[1.43-64.29]	23.57[8.57-43.39]	0.090	0.042	0.910
**PA-Mod (min/day) #**	14.29[6.43-34.29]	12.86[6.07-31.07]	17.14[6.79-37.14]	17.14[8.57-35.36]	13.57[8.57-35.36]	19.29[8.04-35.36]	0.532	0.768	0.734
**PA-Vig (min/day) #**	5.71[0.00-17.14]	4.29[0.00-13.57]	8.57[0.00-29.29]	8.57[2.14-17.14]	8.57[3.21-17.14]	10.71[1.39-17.14]	0.120	0.010	0.603

*SD = standard deviation; # non-parametric; t: Total vs. Total; ♀: boys vs. girls; ♂: boys vs. boys; SBP: systolic blood pressure; DBP: diastolic blood pressure; BMI: body mass index; WC = waist circumference; BMI-Z: BMI z-score; PA-L: light physical activity; PA-Mod: moderate physical activity; PA-Vig: vigorous physical activity.*

In the group with central obesity, the girls had higher SBP (p=0.026), body weight (p<0.001), height (p=0.038) and BMI-z (p<0.001) compared to the group considered adequate. In relation to boys, the group with central obesity showed higher SBP (p=0.002), DBP (p=0.003), body mass (p<0.001) and BMI-z (p<0.001) compared to the group considered adequate ( Table 4 ). However, no differences were identified for the practice of physical activity.

**Table 4:** – Anthropometric variables and blood pressure according to the classification of waist circumference.

	ADEQUATE			CENTRAL OBESITY					

	Total (n=291)	Female (n=163)	Male (n=128)	Total (n=45)	Female (n=10)	Male (n=35)	pt	p ♀	p ♂
**Idade (anos)#**	14.64[13.39-15.94]	14.74[13.64-16.01]	14.49[13.23-15.63]	13.81[12.99-15.52]	13.50[12.95-15.48]	13.94[13.02-15.31]	0.065	0.170	0.326
**Peso corporal (kg)#**	54.50[46.80-61.35]	53.80[46.45-60.80]	55.20[47.58-61.62]	78.40[68.90-89.30]	81.75[70.65-89.38]	78.40[68.80-88.20]	<0.001	**<0.001**	**<0.001**
**Estatura (cm)**	1.62[0.10]	1.59[0.07]	1.65[0.11]	1.66[0.08]	1.64[0.03]	1.67[0.09]	0.005	**0.038**	0.543
**Estatura-z#**	0.45[0.28-0.74]	0.43[0.24-0.68]	0.55[0.32-0.80]	0.74[0.49-0.89]	0.77[0.61-0.90]	0.73[0.46-0.89]	<0.001	**0.005**	0.064
**CC (cm)#**	66.00[61.00-71.50]	64.00[59.00-69.50]	69.50[64.50-72.50]	88.50[84.00-95.00]	81.00[79.00-88.50]	90.00[86.00-97.25]	<0.001	**<0.001**	**<0.001**
**IMC (kg/m2)#**	20.56[18.54-22.77]	21.40[19.13-23.38]	20.11[18.37-21.60]	28.67[26.40-31.40]	29.80[26.30-33.16]	28.67[26.66-30.23]	<0.001	**<0.001**	**<0.001**
**IMC-z**	0.22[1.02]	0.25[1.07]	0.17[0.95]	2.39[0.52]	2.35[0.53]	2.40[0.53]	<0.001	**<0.001**	**<0.001**
**PAS (mmHg)#**	122.50[112.75-131.00]	119.00[111.00-129.75]	124.25[117.50-132.88]	132.00[127.00-139.50]	136.25[129.00-138.25]	131.00[126.75-141.75]	<0.001	**0.026**	**0.002**
**PAD (mmHg)#**	68.50[62.50-74.50]	70.00[65.00-75.75]	67.00[61.00-73.50]	73.00[68.00-78.00]	76.50[68.88-84.38]	72.50[68.25-74.75]	0.009	0.101	**0.003**
**AF-leve (min/dia)#**	19.29[0.00-42.86]	14.29[0.00-38.57]	25.71[8.57-46.61]	21.43[5.71-51.43]	23.57[9.29-57.86]	21.43[5.00-47.14]	0.609	0.432	0.527
**AF-moderada (min/dia) #**	14.29[6.43-35.00]	12.86[6.43-33.21]	17.14[6.43-36.43]	17.14[8.57-34.29]	19.29[6.96-33.75]	17.14[9.64-34.29]	0.377	0.779	0.619
**AF-vigorosa (min/dia)#**	6.43[0.00-17.14]	5.71[0.00-14.29]	8.57[0.00-26.43]	10.71[2.86-17.14]	6.43[0.71-11.79]	12.86[3.57-22.14]	0.271	0.928	0.607

*Abbreviations: SD = standard deviation; # non-parametric; t: Total vs. Total; ♀: boys vs. girls; ♂: boys vs. boys; SBP: systolic blood pressure; DBP: diastolic blood pressure; BMI = body mass index; WC = waist circumference; BMI-Z: BMI z-score; PA-L: light physical activity; PA-Mod: moderate physical activity; PA-Vig: vigorous physical activity.*

Anthropometric and blood pressure measurements were also assessed according to AF-mv ( Table 5 ). In the total sample, active individuals had higher values of body weight (p=0.002), BMI (p=0.016), height z (p=0.001), in addition to lower DBP (p=0.046) compared to those classified as insufficiently active. In relation to girls considered sufficiently active, higher values for body mass (p=0.005), height (p=0.048), WC (p=0.015), BMI (p=0.005) and height z (p=0.016) were identified compared to insufficiently active girls. Sufficiently active boys had higher z-stature (p=0.025) than insufficiently active boys.

**Table 5:** – Anthropometric variables and blood pressure according to the classification of level of physical activity. By sex

	SUFFICIENTLY ACTIVE			INSUFFICIENTLY ACTIVE					

	Total (n=201)	Female (n=93)	Male (n=108)	Total (n=135)	Female (n=80)	Male (n=55)	pt	p ♀	p ♂
**Age (years)#**	14.39[13.17-15.96]	14.48[13.28-15.99]	14.27[13.08-15.88]	14.64[13.56-15.80]	14.84[13.94-15.95]	14.45[13.38-15.10]	0.504	0.442	0.854
**Body weight(kg)#**	58.10[50.50-65.80]	57.40[50.70-63.40]	59.15[50.40-72.30]	53.70[43.90-62.05]	51.60[43.08-60.83]	58.50[48.45- 66.65]	0.002	0.005	0.300
**Stature (cm)**	1.63[0.10]	1.60[0.07]	1.67[0.11]	1.60[0.09]	1.58[0.08]	1.64[0.10]	0.002	0.048	0.114
**Stature z#**	0.56[0.34-0.80]	0.48[0.34-0.73]	0.68[0.35- 0.84]	0.41[0.23- 0.70]	0.38[0.22-0.62]	0.48[0.27-0.71]	0.001	0.016	0.025
**WC (cm)#**	69.50[64.00-75.00]	67.00[61.00-72.00]	71.25[66.38-79.25]	65.00[61.00-72.00]	63.00[59.00-67.00]	70.00[64.00-75.50]	0.001	0.015	0.226
**BMI (kg/m** ^**2**^ **) #**	21.58[19.40-24.50]	22.27[19.69-24.26]	20.98[19.17-24.80]	20.35[18.23-23.56]	20.10[18.25-23.34]	20.39[18.29-24.62]	0.020	0.009	0.530
**BMI**_**- Z**_	0.64[1.11]	0.59[1.01]	0.67[1.19]	0.31[1.34]	0.11[1.26]	0.61[1.41]	0.016	0.005	0.748
**SBP (mmHg)#**	124.50[115.50-132.50]	120.50[112.50-131.00]	129.00[119.12-136.62]	123.00[111.50-130.75]	117.50[108.50-130.62]	124.00[118.00-130.75]	0.067	0.365	0.205
**DBP (mmHg)#**	69.00[62.50-74.00]	70.50[64.50-75.00]	67.75[61.38-73.00]	70.00[64.50-77.75]	70.00[65.00-77.62]	70.00[62.50-77.75]	0.046	0.599	0.063
**PA-L (min/day) #**	34.29[12.86-64.29]	34.29[14.29-64.29]	34.29[12.86-64.29]	8.57[0.00-17.14]	0.00[0.00-12.86]	12.86[2.86-25.71]	<0.001	<0.001	<0.001
**PA-Mod (min/day) #**	25.71[12.86-60.00]	25.71[12.86-60.00]	25.71[12.86-53.57]	8.57[0.00-12.93]	8.57[0.00-12.86]	8.57[0.71-17.14]	<0.001	<0.001	<0.001
**PA-Vig (min/day) #**	14.29[5.71-32.14]	12.86[5.71-25.71]	15.71[8.04-39.64]	0.00[0.00-5.71]	0.00[0.00-4.64]	1.86[0.00-5.71]	<0.001	<0.001	<0.001

*SD: standard deviation; # non-parametric; t: Total vs. Total; ♀: boys vs. girls; ♂: boys vs. boys; SBP: systolic blood pressure; DBP: diastolic blood pressure; BMI: body mass index; WC: waist circumference; BMI-Z: BMI z-score; PA-L: light physical activity; PA-Mod: moderate physical activity; PA-Vig: vigorous physical activity.*

Analyzing the anthropometric parameters CC, BMI and AF-mv with blood pressure ( Table 6 ), the boys presented a higher proportion of prehypertension and arterial hypertension (p=0.033). The individuals considered active had a higher proportion of diastolic prehypertension, while sedentary individuals had a higher proportion of AH (p=0.015). It was observed that adolescents with central obesity, as well as those with overweight, had a higher proportion of prehypertension and hypertension and high SBP (p<0.001).

**Table 6:** – Percentage distribution of blood pressure according to anthropometric values and level of physical activity

		BLOOD PRESSURE			p	SYSTOLIC BLOOD PRESSURE			p	DIASTOLIC BLOOD PRESSURE			p

		Adequate (n=158)	Pre (n=42)	Hypertensive (n=136)		Adequate (n=164)	Pre (n=40)	Hypertensive (n=132)		Adequate (n=287)	Pre (n=18)	Hypertensive (n=31)	
**Sex (%)**	Female	91(52.6%)	15(8.7%)	67(38.7%)	0.033	93(53.8%)	15(8.7%)	65(37.6%)	0.075	145(83.8%)	11(6.4%)	17(9.8%)	0.633
	Male	67(41.1%)	27(16.6%)	69(42.3%)		71(43.6%)	25(15.3%)	67(41.1%)		142(87.1%)	7(4.3%)	14(8.6%)	
**PA-mv (%)**	Suff. Active	91(45.3%)	27(13.4%)	83(41.3%)	0.685	93(46.3%)	27(13.4%)	81(40.3%)	0.412	179(89.1%)	11(5.5%)	11(5.5%)	0.015
	Insuff. Active	67(49.6%)	15(11.1%)	53(39.3%)		71(52.6%)	13(9.6%)	51(37.8%)		108(80.0%)	7(5.2%)	20(14.8%)	
**WC (%)**	Appropriate	151(51.9%)	34(11.7%)	106(36.4%)	<0.001	155(53.3%)	32(11.0%)	104(35.7%)	<0.001	252(86.6%)	16(5.5%)	23(7.9%)	0.103
	Central Obes.	7(15.6%)	8(17.8%)	30(66.7%)		9(20.0%)	8(17.8%)	28(62.2%)		35(77.8%)	2(4.4%)	8(17.8%)	
**BMIz (%)**	Eutrophic	121(55.5%)	25(11.5%)	72(33.0%)	<0.001	125(57.3%)	22(10.1%)	71(32.6%)	<0.001	190(87.2%)	13(6.0%)	15(6.9%)	0.114
	Overweight	37(31.4%)	17(14.4%)	64(54.2%)		39(33.1%)	18(15.3%)	61(51.7%)		97(82.2%)	5(4.2%)	16(13.6%)	

*PA-mv: moderate-vigorous physical activity level; Insuff.: insufficiently; Suff.: sufficiently; Obes.: obesity; WC: waist circumference; BMI z: body mass index z score.*

According to the odds ratio analysis ( Table 7 ), there was no difference between sexes for high AH (OR = 1.40; CI = 0.88–2.22), SBP (OR = 1.35; CI = 0.85–2.14) and DBP (OR = 0.84; CI = 0.40–1.77). On the other hand, according to adiposity indicators, individuals with central obesity were 6.11 (CI = 2.59–14.42) times more likely to have AH than those with adequate WC, while obesity general analysis revealed that the probability of presenting AH is 2.91 (CI = 1.77 = 4.79) higher than those who presented adequate BMI z-score. In addition, it was observed that sufficiently active adolescents had a reduction of approximately one third in the risk of high DBP (OR = 0.33; 95% CI: 0.15–0.72).

**Table 7:** – Odds ratios for the risk of high blood pressure between anthropometric variables and level of physical activity

	HA OR (CI 95%)	PAS elevada OR (CI 95%)	PAD elevada OR (CI 95%)
**Sex=Boys**	1.40(0.88-2.22)	1.35(0.85-2.14)	0.84(0.40-1.77)
**PA-mv= sufficiently active**	1.15(0.72-1.84)	1.21(0.76-1.93)	0.33(0.15-0.72)
**WC= Central obesity**	6.11(2.59-14.42)	4.64(2.10-10.23)	2.50(1.04-6.03)
**BMI-z= General obesity**	2.91(1.76-4.79)	2.75(1.68-4.52)	2.09(0.99-4.40)

*M: male; AF-mv: moderate-vigorous physical activity; WC: waist circumference; BMI-z: body mass index z-score; AH: arterial hypertension; SBP: systolic blood pressure; DBP: diastolic blood pressure; OR: odds ratio; CI: confidence interval; p values with significance level at p <0.005.*

## Discussion

The main results revealed higher risk for the presence of AH in students with abdominal obesity (OR = 6.11) and general obesity (OR = 2.91). In addition, adolescents who practice AF-mv showed a 33% reduction in the risk of high DBP. The current literature has been consistent in showing that BMI-z and WC are strongly associated with AH in childhood and adolescence.^9^ In addition, the findings of this study show a relevant protective factor of the practice of AF-mv for the presence of AH in adolescence, an aspect that has been little explored in population-based studies.

In view of this situation, anthropometric measurements represent relevant predictors of AH, which are justified as a simple, quick, easily interpreted and cost-effective alternative.^24 , 25^ Several reports demonstrate an association between blood pressure, BMI and WC, suggesting obesity as a strong risk factor for the development of AH in adulthood.^9 , 16^ Excessive distribution of visceral fat is accompanied by changes in various inflammatory and endothelial markers,^26^ which stimulate the increase of insulin resistance events, endothelial dysfunction and increased fluid retention, which can then stimulate variations in BP levels and growth of cardiovascular risk.^27^

It was found that 40.5% of the adolescents had AH, with half of overweight students and two-thirds with high WC diagnosed as AH, in a greater proportion in adolescent relationships with adequate measures. In a study of national and regional representativeness that evaluated 73,399 students aged 12–17 years in the southern region of Brazil, the estimated prevalence of AH was 12.5% and that of prehypertension was 17%;excess weight varied between 29.8%^1^ and 35.5%^28^ of South Brazilian adolescents. It is suggested that, in addition to genetic and environmental factors, obesity and AH may be related to metabolic disorders.^27^ Regarding the differences between sexes, a prevalence of AH and similar SBP among boys and girls was identified, however, girls had higher mean DBP. Similar results have been found in the literature.^9^ A possible explanation may the fact that girls practice less physical activities per day compared to boys, which demonstrated a protective effect for high DBP.

In addition, it was observed that overweight girls practice longer physical activities, as well as adolescents considered active had higher averages of anthropometric indicators. This data may reflect participation in physical activities as a strategy to reduce body weight.^14^

It was found that high WC and BMI-z were associated with higher risk of AH, however, those considered sufficiently active showed one-third reduction in the risk of high DBP, which suggests that AF-mv may interfere with blood pressure levels, in addition to reducing metabolic risk.^29^ However, another study^30^ demonstrated that only overweight and obesity were directly associated with AH, but not the practice of AF-mv.^31^ Most adolescents were considered sufficiently active, which may be related to socioeconomic level, as physical activities are offered out of school periods.^32^

The urbanization process, technological advances in modern society and the increase in violence are associated with changes in behavior in children and adolescents.^33^ The increase in time spent doing sedentary activities and less AF-mv practice favor weight gain and diseases associated with obesity, including HA.^1^ At least 300 minutes of AF-mv per week is recommended to provide additional health benefits.^34^

In this regard, an aspect of relevance found in this study refers to the association between lower DBP in adolescents who practice AF-mv, suggesting that the practice of AF-mv may interfere with blood pressure levels in the juvenile population.^30^ A recent study found that adolescents with better muscle skills exhibited lower levels of DBP.^35^ Thus, interventions that encourage the transition from physical inactivity to activity promote immediate impacts on the increase of physical activity among schoolchildren,^36^ which can be considered a protective factor for AH.

Therefore, effectively detecting risk factors early can contribute to the prevention of cardiovascular diseases in adulthood, since changing established habits and attitudes can represent complex tasks and often lead to unsatisfactory results. However, health policies directed at schoolchildren, as well as social investments to improve the practice of AF-mv, may eventually determine significant changes in the population. In this regard, the presence of education and support from health professionals is of great importance, contributing to the control and prevention of AH, among other risk factors associated with cardiovascular diseases.

The present study has some limitations, such as the small sample size, and measurement of blood pressure must be performed on at least three different occasions to better diagnose hypertensive students. Another limitation is the use of a recall questionnaire to assess the level of physical activity, however the IPAQ has an excellent association with AF-mv.^23^ The variables of socioeconomic level, sexual maturity, dietary salt intake and family history of hypertension were not verified either. However, it is noteworthy that the strong point was to associate AH with the diagnosis of central and general obesity, as well as to highlight the importance of the practice of AF-mv as a protective factor against changes in BP in children and adolescents. Such assessments are important as a prevention in public health, as many children and adolescents do not have the opportunity to have their blood pressure assessed at school.

## Conclusions

In this study, it was observed that half of the evaluated students demonstrated AH and one third had general obesity. In addition, the anthropometric measurements of WC and BMI-z were significantly related to the increased risk of the presence of AH, while the practice of physical activities appears as a protective factor of high DBP among children and adolescents. Thus, it is suggested to implement programs that encourage a healthy lifestyle in the school environment, to contribute to the reduction of central and general obesity indicators, as well as to add protection against AH by increasing the practice of AF-mv in the population of children and adolescents.
